# Methods to Improve Molecular Diagnosis in Genomic Cold Cases in Pediatric Neurology

**DOI:** 10.3390/genes13020333

**Published:** 2022-02-11

**Authors:** Magda K. Kadlubowska, Isabelle Schrauwen

**Affiliations:** Center for Statistical Genetics, Sergievsky Center, Department of Neurology, Columbia University Medical Center, New York, NY 10032, USA; mkk2152@columbia.edu

**Keywords:** neurology, diagnosis, unsolved cases, exome sequencing, genome sequencing, long-read sequencing, optical genome mapping, integrative omics, non-Mendelian inheritance, mosaicism

## Abstract

During the last decade, genetic testing has emerged as an important etiological diagnostic tool for Mendelian diseases, including pediatric neurological conditions. A genetic diagnosis has a considerable impact on disease management and treatment; however, many cases remain undiagnosed after applying standard diagnostic sequencing techniques. This review discusses various methods to improve the molecular diagnostic rates in these genomic cold cases. We discuss extended analysis methods to consider, non-Mendelian inheritance models, mosaicism, dual/multiple diagnoses, periodic re-analysis, artificial intelligence tools, and deep phenotyping, in addition to integrating various omics methods to improve variant prioritization. Last, novel genomic technologies, including long-read sequencing, artificial long-read sequencing, and optical genome mapping are discussed. In conclusion, a more comprehensive molecular analysis and a timely re-analysis of unsolved cases are imperative to improve diagnostic rates. In addition, our current understanding of the human genome is still limited due to restrictions in technologies. Novel technologies are now available that improve upon some of these limitations and can capture all human genomic variation more accurately. Last, we recommend a more routine implementation of high molecular weight DNA extraction methods that is coherent with the ability to use and/or optimally benefit from these novel genomic methods.

## 1. Introduction

Etiological diagnosis in pediatric neurological disorders (NDs) and neurodevelopmental disorders (NDDs) is imperative for disease management, counseling, prognosis, treatment, prevention, and quality of life. Due to the lack of powerful diagnostic tools, several parents of children with severe NDs/NDDs often endure years-long diagnostic odysseys of trial-and-error testing with inconclusive results and misdirected treatments. Over the past decade, targeted next-generation gene panels and exome sequencing have emerged as cost-effective ways of identifying the disease-associated variants in Mendelian disorders, including pediatric NDs [1]. Parent-offspring trio sequencing has been proven to be especially effective. It allows for a more efficient variant filtering (based on fitting inheritance models), and it is particularly useful in the context of de novo variant discovery. Although targeted next-generation sequencing, exome sequencing, and genome sequencing can be effective in identifying causal genetic variation in NDs, many cases remain unsolved [2,3]. A recent meta-analysis of clinical gene panel and exome sequencing in epilepsy, autism spectrum disorder, and intellectual disability across 32,331 individuals revealed the diagnostic yields were 17.1%, 24%, and 28.2% respectively (23.7% overall) [4]. Genome sequencing may improve this diagnostic yield up to ~60% [5,6].

The large number of remaining unsolved cases can be attributed to several factors, such as undiscovered genes, non-genetic causes, an insufficient understanding of the functional consequence of variants, and complex inheritance patterns. In addition, a fraction of the unsolved cases can be due to the sequencing techniques used in current diagnostic settings. Although DNA next-generation sequencing (NGS) technology has dramatically improved over the past decades, large parts of the human genome are not interrogated by the current short-read NGS methods used in both diagnostics and novel gene discovery.

This review discusses various methods to increase the diagnostic findings in genomic cold cases with a pediatric neurological condition, i.e., cases which remain unsolved after standard short-read sequencing methods.

## 2. Maximizing the Use of Information in Existing Sequence Data

### 2.1. Extended Analysis of Existing Data

In addition to standard single nucleotide variant (SNV) and insertion/deletion (InDel) detection, additional types of analysis, based on standard short-read massive parallel sequencing data, can lead to a meaningful increase in the diagnostic yield. One effective additional analysis type is copy-number variant (CNV) detection. CNV analysis is estimated by some studies to yield a molecular diagnosis for about 2% of all genetic disorder patients [7]. However, it can be even more essential for patients with NDDs, for whom microarray-based CNV analysis shows that 10–20% carry clinically relevant CNVs [8,9]. This is, thus, particularly useful if no microarray analysis has been conducted. A plethora of computational CNV analysis software is freely available, both for GS (e.g., CNVnator [10] or LUMPY [11]) as well as ES or gene panel data (CoNIFER [12], ExomeDepth [13], and XHMM [14], among many others). Although the lack of a uniform coverage characteristic for ES and gene panels presents a challenge for computational CNV detection, necessitating a critical examination of the software calls and the validation of results by other methods, countless examples of cases solved with CNV detection software have been published (see, for example [9,15]). Some chromosomes and regions appear to be more CNV-rich and are implicated in developmental disorders more than others [16,17,18]. Last, due to the limitations of short reads, orientation and genomic positional information (e.g., is the duplication tandem) for CNV gains is more challenging [19], and nearly impossible to defer from exome data.

In addition to CNVs, other structural variants (SVs; e.g., inversions, translocations, and insertions) can be assessed with NGS data, and breakpoints can be defined [11,20]. Specific algorithms have also been developed to assess mobile element insertions, such as *Alu* and L1 elements [21]. However, difficulties exist to detect some of these SVs, due to the limitation of short-reads, such as limited mappability and a low ability to span SVs. Although the integration of multiple methods (e.g., split-read, read-depth, paired-end, and assembly-based) has improved the identification of SVs in short-read data [22], copy neutral events remain difficult to detect. In addition, many SV breakpoints are flanked by repetitive elements, limiting their detection [23], and more complex SVs, including various rearrangements, may only be partially detected with these methods. Last, it is important to note that these types of SV analyses are mainly restricted to GS data, as exome data and gene panels are limited to their captured region and will miss the majority of breakpoints.

NGS data can also be used to search for runs of homozygosity (ROH) to locate regions of interest, especially when additional family members are available and/or consanguinity is present. Homozygosity mapping is useful to filter down candidate variants to those located in ROH regions. In larger families, the consideration of genetic heterogeneity, including locus and allelic heterogeneity can also be assessed, especially if multiple genes are known to underly the phenotype seen in the family.

Last, a more detailed interrogation of splicing variants other than the canonical ±1 or 2 splice sites can also be helpful [7]. For example, closely located splice region variants, exonic variants, or branch point variants can disrupt wild-type splicing. In addition, splice enhancers, silencers, or the creation/activation of cryptic splice sites may occur far from the canonical splice sites and influence splicing. To improve the identification of some of these variants, the inclusion of genome-wide precalculated splice region variant predictions, such as dbscSNV [24], RegSNPs-intron [25], Combined Annotation Dependent Depletion (CADD)-Splice [26], or spliceAI [27], into the standard bioinformatic pipelines is highly recommended, as they may easily facilitate the identification of important non-canonical splice region variants.

### 2.2. Non-Mendelian Inheritance Models

**Figure 1:**
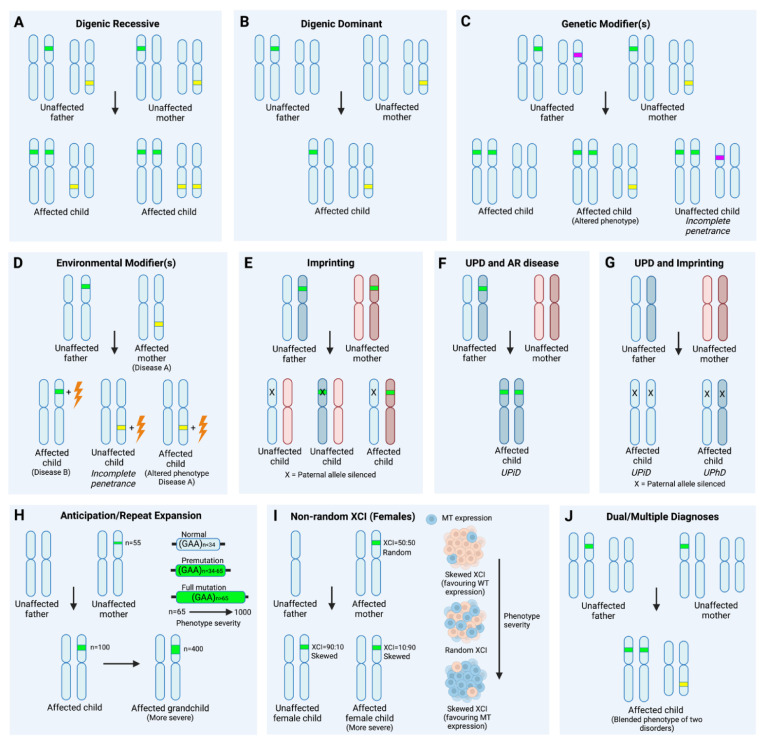
Complex inheritance models to consider in genetically undiagnosed cases, including non-Mendelian inheritance models and dual/multiple diagnoses. (**A**,**B**) Digenic inheritance models, the simplest form of multigenic inheritance. In digenic inheritance, variants at two genomic loci are needed for the manifestation of disease. Digenic inheritance, as classified according to OMIM, can be: (**A**) Recessive, with a biallelic mutant locus 1 together with a variant at a second locus (monoallelic or biallelic at locus 2); (**B**) Dominant, where monoallelic variants at two distinct loci are needed for expression of a disease. (**C**) Genetic modifiers can change the expression of a child’s phenotype and even lead to no observable phenotype (incomplete penetrance). An example of Autosomal Recessive (AR) inheritance is displayed, but modifiers can influence all inheritance models. Genetic modifiers may be rare or common. The presence of multiple rare variants (higher mutational burden) may be associated with a more severe phenotype. (**D**) Environmental modifiers can change the expression of a child’s phenotype and lead to no observable phenotype, i.e., incomplete penetrance (Disease A). In some cases, an environmental trigger is necessary for a phenotype to express (Disease B). (**E**) Imprinting at a certain locus, exemplified here by the silencing of the paternal allele, can alter or lead to the expression of a phenotype. In this example, we assume both parents carry the same variant. If this variant is inherited from the mother, the child will be affected, as the paternal allele is silenced (i.e., both alleles are not functional). If the variant is inherited from the father, this dysfunctional allele will be silenced, and the child will not be affected. In some cases, such as Angelman (MIM# 105830) and Prader Willi syndrome (MIM# 176270), the same variant may lead to different phenotypes, depending on the parent from which it is inherited. Note: variants may also occur de novo. (**F**) Uniparental disomy and AR disease. AR disease may manifest when a child receives two copies of the same copy of a mutant allele, which will occur in some cases of UPD. In this example, uniparental isodisomy (UPiD) of the full chromosome is shown, in which both homologues of a pair of chromosomes from one parent are inherited. Note: UPD may be segmental as well. (**G**) A combination of UPD and imprinting may also lead to disease without the presence of a variant. In this example, the paternal allele is silenced with imprinting. If paternal UPiD or uniparental heterodisomy (UPhD) occurs, both alleles will be silenced, and disease is expressed. Segmental UPD is also possible. (**H**) Repeat expansions can expand over generations, and expanded repeats may lead to the expression of a phenotype or a more severe phenotype (with a longer repeat). (**I**) Non-random X chromosome inactivation (XCI) can alter the expression of disease in female carriers of an X chromosome variant. Expression can be biased towards an increased expression of the mutant allele in a percentage of cells (leading to a more severe phenotype or expression of a phenotype) or wild-type allele (leading to no expression or a milder phenotype). (**J**) Some affected individuals may display a blended phenotype of several disorders. An example here is shown of a child with both an AR variant and a de novo variant. This figure was created with BioRender.com (accessed on 5 February 2022). Abbreviations: AR, Autosomal Recessive; UPD, uniparental disomy; UPhD, uniparental heterodisomy; UPiD, uniparental isodisomy; XCI, X-chromosome inactivation.

Although several non-Mendelian inheritance models, such as X/Y-linked, de novo occurrences, and mitochondrial inheritance, are often considered in the analysis of pediatric NDs, some non-Mendelian inheritance patterns may be more difficult to detect and/or evaluate. Mitochondrial (Mt) DNA NGS sequencing, for example, can be added to ES/GS in the case of suspected mitochondrial inheritance (based on pedigree analysis) or for a suspected mitochondrial disease. Here, we describe a variety of additional and, perhaps more challenging to detect, non-Mendelian inheritance models (Figure 1).

In cases when no single pathogenic/likely pathogenic variant can be identified, more complex disease models can be considered, such as digenic or oligogenic models (Figure 1A,B). Some examples of digenic NDDs include Roifman–Chitayat syndrome (MIM# 613328), AMED syndrome (MIM# 619151), and Joubert syndrome (MIM# 612285, 614464) [28]. In addition, retinitis pigmentosa (MIM# 608133), Usher syndrome (MIM# 605472, 601067), nonsyndromic deafness (MIM# 600791), and short-rib thoracic dysplasia (MIM# 613091, 263520) can also have digenic roots [28]. Due to the difficulty in confirming these types of inheritance in (especially rare) disease, it is anticipated that more oligogenic NDDs exist than there are currently known. Fortunately, computational tools for predicting oligogenic interactions have been developed, which allow the user to submit a variant list or a VCF file to an online interface [29,30] and produce a oligogenic interaction network graph, gene pair ranking, and a classification of variant combinations. Another noteworthy resource is STRING [31,32], a downloadable database of protein interactions.

Genetic modifiers consist of genetic variation outside of the disease-associated genes influencing the expression of disease or altering its phenotype (Figure 1C). Some genetic modifiers can have a protective function and lead to incomplete penetrance. These are known as suppressors. Otherwise, they can lead to a more severe disease manifestation or alter the disease presentation [33]. For example, deafness caused by homozygous pathogenic variants in *GAB1* appears to be suppressed by the presence of a heterozygous variant in *METT13* [34,35]. Alternatively, genetic modifiers may increase phenotype severity, which may increase further with the presence of multiple rare variants, also often referred to as a higher “mutational burden”. It has been suggested that females are more resilient to a higher mutational burden than males in NDDs (unlinked to chrX) [36]. While the awareness of and interest in genetic modifiers is increasing, it is important to keep in mind the difficulty of studying this phenomenon in rare disease. The small number of patients (in some cases conclusions are drawn from a single family) limits researchers’ ability to draw statistically sound conclusions. Staying abreast with the latest research and, when possible, considering variant segregation within a proband’s family are recommended when the possibility of modifier involvement arises in a diagnostic setting.

Similar to genetic modifiers, incomplete penetrance or altered disease phenotypes may also be due to environmental modifiers (Figure 1D). This is not unexpected in neurodevelopmental disease, as environmental factors, such as dietary intake during pregnancy, have been shown to affect fetal development [37]. Infections and drug treatment can also significantly impact child development and the development of disease [38]. In addition, both environmental and genetic modifiers and the interaction between them may alter disease course and manifestation in a complex setting. For example, variants in *SCN1A* are often reported to have incomplete penetrance and broad phenotypic variability [39], which may be due to both genetic and environmental modifiers [40].

Imprinting may also contribute to non-Mendelian disease manifestation (Figure 1E,G). For example, a pathogenic variant may only be expressed and cause disease when maternally inherited, due to imprinting at this locus. When the same variant is inherited from the father, the mutant allele is silenced, and no disease is manifested.

Uniparental disomy (UPD) is another mechanism of non-Mendelian inheritance (Figure 1F,G) [41] that can lead to disease in autosomal recessive disorders (Figure 1F) or through imprinting (Figure 1G). The most recent estimate of the occurrence rate of UPD in the general population is 1 in 2000 [42], although individuals with severe phenotypes were likely underrepresented in this estimate [43]. There are two types of UPD: isodisomy (UPiD), where two identical copies of a chromosome/part of chromosome inherited from one parent replace the allele from the other parent, and heterodisomy (UPhD), where two non-identical chromatids are inherited from one parent and none from the other. Isodisomy can be relatively easily detected by searching for ROHs; when discovered, it suggests an increased likelihood of an autosomal recessive disorder. Heterodisomy is more common, but individual ES data is insufficient to diagnose it. However, this can be detected easily via parental DNA sequencing.

Maternal or paternal UPD can have clinical consequences through imprinting, without the presence of a pathogenic genomic variant (Figure 1G) [41]. Examples of syndromes that can be caused via UPD and imprinting include Temple (MIM# 616222), Kagami-Ogata (MIM# 608149), Silver–Russell (MIM# 180860), Prader–Willi (MIM# 176270) and Angelman syndromes (MIM# 105830). The establishment of maternal or paternal UPD can, therefore, be important in disease etiology. Alternatively, an analysis of the methylation status of the chromosomal regions containing imprinted genes [41] is one possible testing strategy. If heterodisomy of chromosomes or differentially methylated parts of chromosomes 6, 7, 11, 14, 15, or 20 are diagnosed, the possibility of an imprinting disorder should be interrogated. Heterodisomy of other areas may be harmless [41]. A mixture of isodisomy and heterodisomy (created as a result of recombination) is often observed [44], and advanced parental age appears to be a risk factor [43]. UPD can arise via a variety of mechanisms, some of which (such as trisomy or monosomy rescue) can lead to mosaicism. For this reason, the discovery of UPD should prompt a search for mosaicism.

Last, in disorders of repeat expansion (most famously, fragile X syndrome (MIM# 300624), but over fifty such disorders are known, some linked to loci outside the exome) the phenotype can emerge earlier or become more severe if the number of repeats grows from one generation to the next (anticipation) (Figure 1H) [45,46]. Phenomena such as non-random X inactivation in females can also alter the phenotype seen in females in X-linked disease, ranging from severe to no phenotype (incomplete penetrance) (Figure 1I). Finally, some exceptional dominant variants, such as in glaucoma-associated *MYOC* and cognitive impairment due to *PCDH19*, have been found to be heterozygote-specific, with homozygous/hemizygous individuals remaining asymptomatic or less severely affected, a phenomenon aptly described as paradoxical inheritance [47,48,49].

### 2.3. Dual/Multiple Diagnoses

In a situation where a variant fully matching the patient’s phenotype cannot be identified, a blended phenotype may be considered (Figure 1J). It is estimated that, on average, multiple (two or more) diagnoses apply to about 4% of cases solved by ES [50]. While investigating the incidence of multilocus variation in a cohort of 108 neurodevelopmental patients, Karaca et al. established that multiple diagnoses applied to 12% of the families. Of note, this included 6 of 19 families previously deemed to show a phenotypic expansion [51]. Posey et al. described a large cohort of 7374 patients and found a multiple diagnosis in 4.9% of those with a molecular diagnosis. In total, 44.7% of all multiple diagnoses patients had 2 de novo causative variants [52]. Although co-occurrence of rare disease appears intuitively unlikely, Lal et al. show that the probability of a single individual carrying pathogenic variants in more than one rare disease gene goes up significantly when consanguinity is involved [53]. This being said, a blended phenotype should also be considered when one of the family members shows a markedly more severe phenotype [51]. Seemingly syndromic cases that were eventually explained by variants in multiple different genes have been described [54,55,56]. In a rare scenario, Li et al. report on a case of siblings with congenital hypothyroidism, hypomagnesemia, and hypercholesterolemia that were suspected to suffer from a new disorder but were eventually diagnosed with three different autosomal recessive disorders [55].

### 2.4. Mosaicism

**Figure 2:**
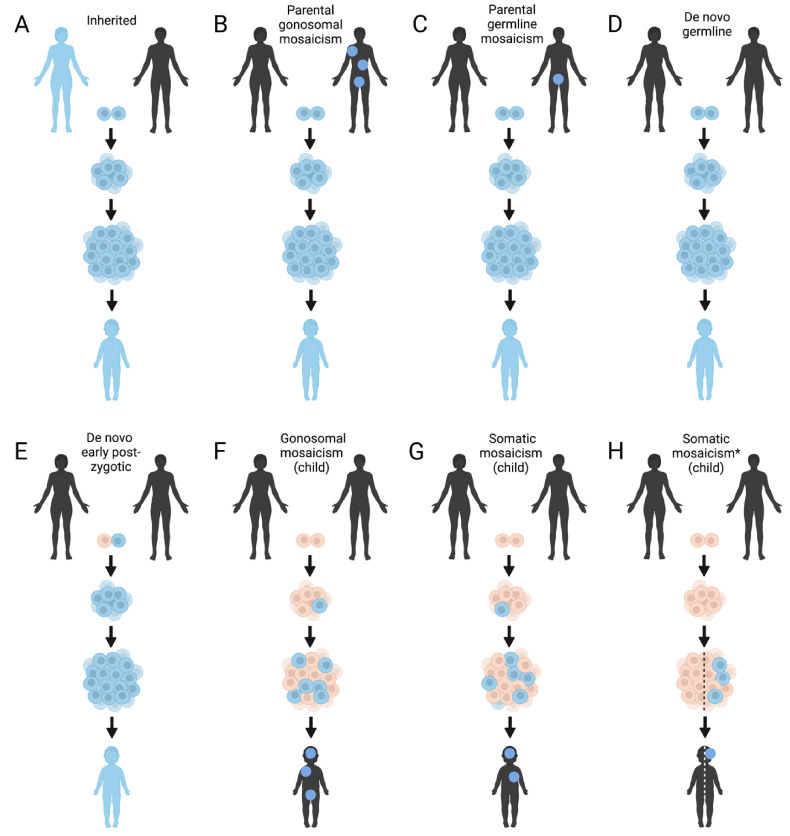
The different levels of mosaicism in Mendelian diseases and how they manifest. (**A**) An autosomal dominantly inherited variant that is present in all cells of the parent and child. (**B**) In gonosomal mosaicism, the variant is present within both the germline and somatic cells. The parent here with gonosomal mosaicism of a variant may express a phenotype, depending on the tissues affected. If the child inherits the variant, it will be present in all cells. Multiple children of this parent can be affected. (**C**) Germline mosaicism, also called gonadal mosaicism, only occurs in the germ cells (gametes). The parent will not be affected with disease (with the exception of, perhaps, infertility). If the child inherits the variant, it will be present in all cells. Multiple children of this parent can be affected. (**D**) With a de novo germline variant, a single germ cell is affected with the variant, and all cells of the affected child will have the variant. In this case, reoccurrence of this disorder in additional offspring is unlikely. (**E**) In cases of very early post-zygotic mutations, all cells of an affected child can be affected. This is because not all cells in the early development (before the blastocyst stage) contribute to the embryo. In this case, reoccurrence of this disorder in additional offspring is unlikely. In practice, it is difficult to distinguish between (**C**–**E**), and often also between (**B**–**E**) if the parent is asymptomatic. Most of (**C**–**E**) will be referred to as “de novo*”*, however in **B**/**C**, there is a chance of recurrence in multiple offspring. In (**F**) a gonosomal mosaic variant is presented, which occurred de novo later in development. The phenotype of the affected child will depend on the tissues of presence; however, the variant may be transmitted to the next generation, as it is present in the germ cells. In (**G**) a somatic mosaic variant is presented. The phenotype of the affected child will depend on the tissues of presence; however, the variant will not be transmitted to the next generation, as it is not present in the germ cells. The same is presented in (**H**). However, in this case, the variant arose after left-right determination, affecting only tissue(s) on one side of the body. * Also possible for gonosomal mosaicism. This figure was created with BioRender.com (accessed on 5 February 2022).

Mosaicism is known to be a factor in a range of pediatric NDs (Figure 2), including autism (estimated to contribute 3–5% of simplex ASD risk), Cornelia de Lange syndrome (MIM# 122470), Proteus syndrome (MIM# 176920), Sturge–Weber syndrome (MIM# 185300), MCAP syndrome (MIM# 602501), and some epilepsies [28,57,58,59,60,61,62]. It is also likely responsible for up to 30% of cases in disorders of neuronal migration [63]. In addition, mosaic aneuploidy (present in neuronal and other tissues) has been shown to occur in some patients with Down syndrome (MIM# 190685), Seckel syndrome (MIM# 210600) [64], and in several other rare NDDs. It is worth noting, however, that mosaic aneuploidies, CNVs, and SNVs have also been detected in the normal adult brain, and that hundreds of somatic SNVs are already present at birth [63,65]. In general, three main types of mosaicism can be defined: (1) germline mosaicism (variant present in germ cells but not elsewhere in the body), (2) somatic mosaicism (variant present in some of the somatic cells but not germ cells), and (3) gonosomal mosaicism (mosaic variant is present in germ cells and some of the somatic cells) [63]. In practice, it is sometimes difficult to distinguish between these classifications and when exactly a variant appeared de novo in development in the parents and/or child (Figure 2). The number of tissues and/or cells affected by mosaicism depends on the stage of development at which the mutation arose; events occurring later can be expected to result in a lower variant allele fraction (VAF), to be more localized and to be harder to detect, while even a VAF of 1% can result in a disease phenotype [66].

The accuracy of detecting mosaicism with NGS depends on the NGS coverage depth, the tissue used, and the presence of controls and/or parental samples. While some ND-causing somatic mutations can only be found in brain tissues [66], skin biopsies [67], cultured fibroblasts [67], buccal swabs [67], saliva or even blood can be used, although with a lower likelihood of detecting mosaic variants [67,68,69]. Since buccal mucosa cells also originate from the ectoderm, similar to the nervous system, in contrast to peripheral blood leucocytes (mesoderm), mosaic variant detection in NDs may be preferred in buccal swabs [70]. The DNA extracted from saliva is derived from both buccal epithelial cells and leucocytes. However, direct buccal swabs contain a higher proportion of epithelial cells [71]. Although more invasive, skin biopsies may also deserve consideration in cases of suspected mosaicism in NDs, as the epidermis of the skin originates from the ectoderm. A comparative study showed, however, that non-invasive buccal cells showed a high diagnostic sensitivity in the detection of mosaic variants for diseases of the brain and head, with similar rates compared to invasive tissue types, such as skin biopsies [67]. Nevertheless, skin tissue is also important to consider in neurocutaneous disorders, and in a number of cases, skin abnormalities even show a visible mosaic pattern [72]. Note that fibroblasts cultured from skin biopsies (dermis) originate from the mesoderm. However, culturing may increase the allelic fraction of variants that have a growth advantage [67].

In clinical practice, the lower boundary of detecting mosaic variants via ES is approximately 10–20% with sufficient average coverage (>100×) [73,74]. The difficulty of detecting somatic mutations with NGS stems primarily from the difficulty of distinguishing sequencing artefacts from low VAF mutations [75,76]. Moreover, the most commonly used variant callers have been shown to be ill-suited for calling somatic variants with VAF <10%, even for 250× GS data [75,76]. Still, computational tools for mosaicism detection are available and are used (e.g., MrMosaic [77], MosaicHunter [78], and MosaicForecast [79]), though due to the high rate of false positives, they require careful validation, e.g., by ultra-deep sequencing or with droplet digital PCR (ddPCR), which can detect VAFs as low as 0.1% and 0.001% respectively [75]. A comprehensive pipeline for somatic variant detection from GS data has recently been made available by the Brain Somatic Mosaicism Network (BSMN) [76]. The pipeline is based on BSMN-defined best practices for somatic variant detection and implements tools, such as 1000 Genomes Strict Mask (to remove low mappability regions) and panel of normals (PON) filtering to remove other commonly occurring technical artifacts, amongst others [76].

If a putative germline de novo variant is suspected to be disease causing, parental mosaicism may be considered, especially due to its impact on the risk of recurrence and, hence, genetic counseling [68]. Unlike germline de novo variants, parental mosaicism does not seem to correlate with parental age [68,80]. It has been shown that paternal sperm mosaicism assessment can be of value in providing a more accurate recurrence risk [80].

## 3. Data Re-Analysis

### 3.1. Periodic Data Re-Analysis

Difficulties in diagnosing cases of suspected genetic disease based on an analysis of ES/GS data may stem from gaps in current knowledge. Multiple studies reporting on the diagnostic yields of the re-analysis of sequencing data list the increased understanding of gene–disease associations and variant pathogenicity as the primary causes of successful diagnosis upon re-examining patient ES/GS data [81,82]. The recommendation as to the length of time after initial analysis when re-analysis should be carried out vary from group to group, but many studies report diagnostic yields up to 30% for cases examined 18–24 months after the initial analysis [81,82,83]. The American College of Medical Genetics and Genomics (ACMG) notes that re-analysis might also be warranted if a new resource (e.g., a new database or new variant interpretation guidelines) or new patient phenotype information become available [84].

The most common re-analysis approach consists of reannotating the data [82]. However, the use of a new/different bioinformatics pipeline may increase the likelihood of successful diagnosis [85]. In cases where only singleton ES was carried out, extending the testing to a trio analysis or WGS should also be considered [84,85]. In addition, in some cases, realigning to a new reference build of the genome can uncover new, potentially causative variants [86,87].

A recognized obstacle to periodic ES re-analysis is the significant workload involved. In an attempt to address this challenge, efforts in automating the process and extending the use of computational tools have been described, with some reported success [88,89]. Such tools can include, for example, literature and database mining [88] or the use of dedicated artificial intelligence (AI) diagnosis-support solutions [89].

Last, listing potential candidate genes on platforms such as ModelMatcher and GeneMatcher [90] allows researchers to connect to others who may be interested in or are already studying a particular gene in vitro and/or in vivo, and further follow-up may identify novel genes implicated in disease.

### 3.2. Artificial Intelligence (AI) Applications

Medical applications of artificial intelligence (defined broadly as the development of machines or programs displaying intelligent behavior) and, specifically, machine learning (a subfield of AI) enjoy an ever-increasing interest, as illustrated by a growing number of publications [91]. The use of AI in a diagnostic context can be informally divided into the following categories: (1) variant deleteriousness prediction [92], (2) intelligent search engines (providing diagnosis suggestions based on user-entered clinical data, e.g., FindZebra [93,94] or PubCaseFinder [95]), (3) image-based evaluation (such as Face2Gene, which uses face recognition technology to provide a list of syndromes matching a patient’s appearance, or AI tools assisting in the diagnosis of ASD based on MRI images [96]), and (4) variant ranking based on NGS data and phenotype. The latter comes in two categories: as freely available tools that may require some minimal amount of bioinformatics skill (e.g., Java-based Exomiser [97,98] or R package Xrare [99]) and as user-friendly commercial solutions (e.g., the Genoox, Emedgene, or Moon platforms). In what follows, we briefly describe some of these tools, referring the reader to cited literature for more detailed information.

Exomiser was first presented in 2014 [97] and has been updated many times since. Reports of successful Exomiser-assisted diagnoses can be found in the literature (e.g., [100,101]), and a recent benchmarking test of Exomiser version 12.0.1 on a cohort of 134 monogenic disease cases reports that for 96% of these patients, the true causative variant was listed within the top 10 selected by the software [98]. Exomiser takes in a VCF file and a Human Phenotype Ontology (HPO) phenotype description and applies filters and multiple variant pathogenicity prediction tools to rank the phenotypic fit using a semantic similarity method. The output is a prioritized list of variants. Another tool from the same class, Xrare, uses a different similarity scoring system so as to be tolerant of noisy and imprecise phenotype descriptions [99].

The tools described above are two examples among many diagnosis support programs available free of charge. Commercial solutions in this field have also emerged, offering to alleviate the hassle of environment setup, an online graphical user interface backed by ongoing bioinformatics support, and continuity of updates—things that may be difficult or impossible to provide for academics developing free software. The increasing popularity of commercial tools is reflected in the literature (e.g., [102,103,104]). One of the more commonly mentioned platforms is Genoox, which offers a user customized pipeline handling everything from alignment and variant calling to diagnosis support provided by an AI engine named Franklin. The tool has been successfully used both for solving new cases and for periodic re-analysis [89,105,106,107] and is now introducing an integration with optical genome mapping data (Bionano, San Diego, CA, USA) to enable the analysis of structural variants [SVs].

## 4. Integrating Omics to Understand Functional Effects and Improve Variant Prioritization

Prioritization of candidate variants remains challenging, mainly due to an insufficient understanding of the functional consequences of a substantial fraction of variants. The disease-causing variants might be detected by ES/GS but remain as variants of unknown significance (VUS) [108], especially when in a non-coding region. The integration of multiple omics data can be helpful in prioritizing VUS variants.

### 4.1. Transcriptomics

Simultaneous DNA and RNA sequencing allows the immediate evaluation of in silico bioinformatic predictions on the effect of genomic variants on gene expression, alternative splicing, etc. RNA-seq can aid in both the identification of novel variants and genomic variant prioritization. It can provide evidence of pathogenicity for variants that were not found or were unsuspected via DNA sequencing (e.g., intronic cryptic splice site variants). In addition, it can corroborate the effect of suspected variants and provide further insight into likely pathogenic/pathogenic variants, such as the level of nonsense-mediated decay caused by loss-of-function variants, and the impact of this loss on other genes within the same pathway [109].

By supplementing DNA data with RNA data, researchers can (re-) prioritize rare genomic variants by complementing them with an outlier score or detect novel events not found in DNA data. These analyses can focus on identifying (a) aberrant splicing; (b) aberrant expression; and (c) allelic imbalance, including non-random X-inactivation. Non-random X-inactivation can be evaluated by assessing allelic expression over the entire X-chromosome in females. Non-random inactivation is commonly implicated in X-linked neurodevelopmental diseases, and assessing this can be informative in both gene discovery and in diagnosis [110,111,112].

Supplementing DNA-sequencing data (either ES/GS) with RNA-seq can substantially improve molecular diagnosis, which ranges from 10–35% in Mendelian disorders [2,108,113]. Previous studies have successfully used RNA derived from cultured fibroblasts from patients, muscle tissue, and blood samples in the diagnosis of various Mendelian disorders [2,108,113]. Supplementing DNA-sequencing with blood-derived RNA-seq can increase the success rate of gene/variant identification by 16.7%, based on previous studies [113]. This study also showed that for five cases with neurological disorders where blood is not assumed to be a representative tissue, candidates were identified [113]. On another note, of the disease genes from the Online Mendelian Inheritance in Man (OMIM) database, 70.6% were found to be expressed in whole blood samples via RNA-seq. In addition, of the genes implicated in neurological disorders, 76% were expressed in blood, and of genes that are intolerant to loss-of-function, 66% were expressed in blood [113]. Last, variants that have more severe consequences occur more often in genes for which expression is not restricted to one tissue [113]. This all highlights the utility of whole blood mRNA-seq, even for the evaluation of transcripts primarily expressed in other tissues. The overall insight gained from mRNA-seq from whole blood is encouraging, as blood samples have the advantage of being easily accessible in clinical practice; therefore, this can be easily implemented into standard diagnostic practices.

### 4.2. Epigenomics

The term epigenomics refers to the analysis of epigenetic modifications (e.g., DNA methylation, chromatin accessibility, histone modifications, and the three-dimensional (3D) arrangement of DNA) affecting gene expression. It has been found that a disproportionately large number of genes linked with NDs code for proteins involved in the regulation of the epigenetic state [114,115]. Less commonly, variants in genes not directly involved in epigenetic machinery can have an effect on the epigenetic code and methylation in particular [116]. Moreover, larger SVs can affect the 3D architecture of the genome, leading to changes in gene expression. Finally, disease-causing epigenetic changes (e.g., epimutations) may arise without the presence of pathogenic genomic variants, as is sometimes the case in imprinting disorders (Figure 1G) [114]. All this makes epigenomics a suitable complement for more traditional genetic testing.

The two testing modalities that have been most successful in diagnosing NDs are methylation analysis (local and global) and high-throughput conformation capture (Hi-C). DNA methylation, the addition of a methyl group to the carbon 5 of cytosine: 5-mC, is the best characterized and most stable epigenetic mark. Testing methylation levels at disorder-specific regions is currently used in clinical practice to confirm a suspected fragile X or imprinting disorder diagnosis [117]. Rapid developments are being made in the area of genome-wide methylation analysis, which now allows for the identification of the “episignatures” (methylation patterns that have been associated with disease) of over 40 disorders, most of them syndromic NDDs [118]. Work on expanding this list continues, especially in Canada, where a machine-learning-based method named EpiSign is undergoing a national trial aimed at evaluating the clinical utility of this test and also at expanding the list of known episignatures [119]. At present, episignatures can be used to evaluate VUS detected in genetic testing or to confirm the suspected diagnosis in cases with a clear phenotype where DNA sequencing failed to detect the causative variant [117,119].

More recently, cytosine hydroxymethylation (5-hydroxymethylcytosine; 5-hmC) has also gained attention as another relatively stable epigenetic DNA modification [120]. 5hmC is oxidized from 5mC through the ten-eleven translocation (TET) family of enzymes and has a remarkably high presence in the brain [121]. For example, MeCP2, implicated in Rett syndrome (MIM# 312750), is an important 5hmC-binding protein in the brain [121]. It is important to note that certain techniques, such as sodium bisulfite conversion approaches, cannot distinguish 5-mC and 5hmC and, therefore, analyze both epigenetic modifications.

Another important epigenetic regulator of gene expression is the 3D folding of chromosomes, which can bring distant regulatory and functional elements into close spatial proximity. Hi-C is a technique for probing that 3D structure through a process of crosslinking neighboring regions, digesting the DNA with a restriction enzyme, ligating crosslinked fragments, and then sequencing them. Hi-C can be used to evaluate the impact of larger SVs, especially ones occurring in noncoding regions or on topologically associating domains [TADs]. Disruption of TADs, such as, for example, a TAD fusion resulting from a deletion, or the formation of new TADs caused by a duplication, can lead to the gain or loss of an interaction between enhancers and promoters, which in turn can change gene expression and potentially lead to disease. A recent study has shown that Hi-C can help interpret the effect of SVs by the identification of fused-TADs promoting ectopic enhancer–promoter interactions [122]. Together with dedicated software that allows for the easy visualization of the resulting data, Hi-C data can not only expose potential changes to the enhancer–promoter interaction but also help elucidate the structure of more complex SVs [122,123].

While the applications of episignature analysis and of Hi-C in the field NDs diagnostics are relatively new, the increasing popularity of both is to be expected. With epigenomics still being a relatively young field, rapid advances in both technology and knowledge promise to shed more light on the mechanisms of pediatric NDs.

### 4.3. Proteomics

Proteomics is commonly defined as the large-scale analysis of the protein content of biological samples. Over the past two decades, its methods have undergone significant expansion and improvement, allowing for the study of protein conformation, function, interactions, and posttranslational modification, among others [124,125]. In the field of ND research, proteomics has been employed in the quest for biomarker discovery (notably in autism) and in the study of the molecular mechanisms of disease [126,127,128,129,130]. In diagnostics, targeted proteomics can assist in assessing the consequences of the genetic variants discovered by NGS [131]. The fact that transcripts from neighboring genes are often found in similar abundance, while at the level of protein the correlation no longer holds, can serve as evidence that protein levels cannot always be determined with sufficient accuracy based solely on the number of mRNA transcripts present in the cell [132,133]. An interesting example, and one that underscores the importance of protein degradation in establishing protein levels, is the fibroblast proteome of trisomy 21, where expression dysregulation also affects proteins not linked to chromosome 21, and the correlation between steady state chromosome 21 proteins and transcripts levels is moderate, while the fold change correlation was found to be weak [134]. In light of this, one can appreciate the potential of proteomics in assessing the impacts of CNVs, novel variants in untranslated regions, splice sites, possible nonsense mediated decay, and of VUSs in general [131,135].

### 4.4. Metabolomics

Metabolomics, the study of the small molecule content of biological samples, is a relatively young area of research, yet it appears to hold great promise, both in the area of biomarker discovery and in diagnostics. In the latter domain, it is primarily seen as a complement to genomics, since it can be used to prioritize rare variants detected by NGS (untargeted metabolomic) or provide evidence for the pathogenicity of VUSs (targeted or untargeted metabolomics) [136,137,138,139,140]. Metabolomics tools can also be used to study the exposome (the sum total of lifetime exposures starting from conception). For example, in utero exposures to heavy metals and phthalates, which can impact development, have also been found to leave a mark on the metabolome, in particular the pathways related to the tricarboxylic acid cycle and oxidative phosphorylation, possibly due to disruption of mitochondrial respiration [141]. While metabolic abnormalities have been found in patients with such NDDs as ASD (an increase in lactate and creatine and a decrease in creatinine are among many suggested changes that have been reported [127,139,142,143,144]), Rett syndrome (MIM# 312750; metabolites associated with urea and the Krebs cycle and the metabolism of certain amino acids [145]) and Down syndrome (MIM# 190685; alterations to methylation metabolism, carnitine/O-acetylcarnitine, dimethyl sulfone, and myo-inositol [143]), the group of disorders where metabolomics has the most obvious diagnostic application is that of inborn errors of metabolism (IEM), affecting 1:1000 to 1:2000 newborns [137,146]. To date, there are 1615 known IEM and over a half of them show neurologic involvement, including at least 231 presenting with a movement disorder and at least 116 treatable IEMs causing intellectual disability [147,148,149,150,151]. It has also been suggested that ASD patients, at least those born outside of high income countries, are underdiagnosed for IEM [148]. An online tool, Treatable ID App, has been created to assist clinicians in selecting a targeted metabolic workup. However, there are also advocates of using an untargeted approach to maximize the scope of diagnosable disorders [137,146].

Although the importance of metabolomics in pediatric ND diagnostics will grow with the progress of biomarker research, it is important to remember that the metabolome is sensitive to changes in diet, medication, sex, age, sample handling, and other factors. Because of this, a negative metabolomic result cannot always be taken as conclusive.

### 4.5. Public Recourses and Bioinformatic Predictions

In an era of increased data sharing, many datasets are now available to researchers to help prioritize the variants and genes implicated in NDs. For example, the Genotype-Tissue Expression (GTEX) project [152], which studies tissue-specific gene expression and regulation in the context of genomic variants, can be helpful to identify expression in tissues of interest and to identify functionally relevant genomic regions (e.g., intronic).

In addition, single cell RNA sequencing (scRNA-seq) and/or single nuclei sequencing have significantly progressed the characterization of the cellular diversity in the human brain and nervous system. This technology has also helped characterize the trajectories of progenitor maturation and neurogenesis in the developing brain, which may be critical in the development of NDDs. Important trajectories related to a gene’s function can be identified in scRNA-seq data by detecting the timepoints and cell types with increased gene expression. Many datasets, including the human developing brain [153,154], the adolescent mouse brain and nervous system [155], and the adult human and mouse cortex scRNA-seq datasets from the Allen Brain Atlas [156], are publicly accessible via the UCSC cell browser [157]. These datasets are particularly helpful in the context of novel gene discovery in unsolved cases.

Next, the interaction between proteins and biological pathways can be studied using public datasets, including the BioGRID [158], STRING [31,32], and Reactome databases [159]. In pediatric neurological disorders, the expression of 24–29% of the affected protein’s close interactors can be affected [109,160], information which may also be useful in gene discovery.

Last, there are several bioinformatic prediction scores that are helpful to evaluate non-coding variants that integrate various publicly available functional genomic annotations (e.g., ENCODE), such as CADD [161], Genome Wide Annotation of VAriants (GWAVA) [162], and Eigen [163]. Deep learning–based sequence analyzer (DeepSEA), for example, utilized public large-scale chromatin-profiling data to train its model and allow predictions of the effect of variants on various regulatory features, such as transcription factor binding, DNase I sensitivity, and histone marks [164].

## 5. Deep Phenotyping

Deep phenotyping can be defined as an exhaustive, precise description of phenotypic traits, a description that may include the age of onset of each of patient’s symptoms, as well as patient’s test results in the form of text and/or images [165]. While large databases of minimally phenotyped data have led to many genetic discoveries, detailed phenotyping may, in some cases, allow for insight in spite of the limited amount of data characteristic of rare disease [166]. Deep phenotyping can do more than assist in diagnosis by narrowing down the list of potential causative variants; when the result of NGS is a previously undescribed variant or VUS, a close match between the complex phenotype of the patient and a disease associated with the gene in which the variant is located can serve, to some degree, as evidence of causality. While the diagnostic value of ES for NDs is great, the technology is not immune to error. Pena et al. described cases of patients with a compelling phenotype yet negative ES results for whom the suspected diagnosis was confirmed by Sanger sequencing and/or multiplex ligation-dependent probe amplification (MLPA). In some, though not all, cases, such false negative results could be refuted by the manual inspection of BAM files—a practice only possible when there a strong suspicion of a disease associated with one or few genes [167].

While the desirability of a detailed, accurate description of patient’s phenotype seems obvious, its appreciation is not always good enough to outweigh the cost. Imprecision in health records is not uncommon [99], and dedicated deep phenotyping tools have been developed to help avoid it going forward (e.g., [168,169]). Deep phenotyping is also important for successful discovery of new syndromes and new gene–disease associations with the aid of data sharing platforms, such as Matchmaker Exchange [170].

## 6. Novel DNA Sequencing and Mapping Technologies

Current short-read NGS technologies fail to give a complete picture of the human genome. Despite the use of state-of-the-art bioinformatic algorithms, it is often impossible to accurately map, or even assemble, short reads originating from regions with SVs, highly homologous regions, repetitive sequences, or regions with high GC content within the genome [171]. Recently, novel methods have emerged that can better assess regions with high homology and repetitive regions, which encompass the majority of the human genome (i.e., the “dark matter”). In addition, these methods can more reliably detect complex variants, which often go undetected with conventional next-generation sequencing methods. Some of these technologies include long-read sequencing, artificial long-read sequencing, and optical genome mapping [172]. Various studies using these newer technologies have reported that approximately 20 K SVs per human genome exist, most of which could not be detected using short-read sequencing [172].

### 6.1. Long-Read Sequencing (LRS)

Long-read sequencing, often referred to as third-generation sequencing, includes the assessments of long-reads of DNA (>10 kb), originating from single DNA molecules. These technologies measure DNA sequences in real-time, and there is no PCR amplification, which would introduce bias. Due to its ability to retain long-read information, it can be used to more accurately assess complex variants, assess NGS “dead zone” regions with high homology, assess repeat expansions, assess regions with high GC content, and phase the human genomes into haplotypes.

Two main long-read sequencing technologies are available, developed by Pacific Biosciences and Oxford Nanopore. The first technology, developed by Pacific Biosciences, is single-molecule real-time (SMRT) sequencing. It is a single molecule DNA sequencing method based on the real-time recording of changes in light emitted from the nucleotides incorporated in base elongation. Currently, there are two main sequencing options, CLR mode (continuous long read) and CCS mode (circular consensus sequencing). CLR mode allows the sequencing of long reads measuring 25–175 kb. However, the accuracy is reduced, resulting in an error rate of 8–15% [173]. SMRT Sequencing technology has more recently evolved to a second type of long read (CCS mode), known as high-fidelity (HiFi) long reads. These reads have improved single-base accuracy, e.g., an error rate of 1% or less, similar to Illumina short-reads, but are restricted in size to 10–20 kb [173].

The Oxford Nanopore Technologies platform uses nanopores to measure a nucleic acid sequence in real-time through changes in the ionic current across a membrane as a single DNA molecule passes through this protein nanopore [174]. Mainly used for bacterial and viral genomes, recent changes in throughput have allowed for high enough coverage to make sequencing of the human genome feasible with this technology. The Oxford Nanopore technology can generate ultra-long reads, ranging from 500 bp to a record of 2.3 Mb. It is limited only by the integrity of the DNA extracted, with 10–30 kb genomic libraries being common [175]. However, it has high error rates (5–20%) [176] that are dominated by false deletions and, in particular, homopolymer errors [171]. Last, this technology has emerged as a portable technology, with a pocket-sized MinION device as the portable option.

Since these technologies interrogate long spans of DNA, they require high molecular weight (HMW) DNA as an input, which may not always be available, especially for archived samples. Another limitation of these technologies is the current high price point. Long-read sequencers are, therefore, unable to compete with the sequencing depth of the short-read approaches. In addition, there is limited single base pair accuracy, with the exception for HiFi reads, which is coupled with the significant reduction in read size. Other applications of LRS include high-quality transcriptome information, detecting alternative splicing isoforms, epigenetic modifications, and more.

In medical genetics, LRS approaches have so far mainly been used in a targeted setting, e.g., to investigate genetic disorders with previously known, strongly suspected disease loci, or to reconstruct identified SVs [171]. For example, LRS was able to fully reconstruct chromothripsis, a chaotic and complex genomic rearrangement, in a patient with Langer–Giedion syndrome (MIM# 150230) and Cornelia de Lange syndrome (MIM# 614701) [177]. However, some studies have successfully implicated genome-wide long-read sequencing in the discovery of complex SVs in Mendelian neurological disorders [178,179,180], including transposon-mediated events and complex SVs.

### 6.2. Artificial Long-Read Sequencing (Alrs)

Artificial long-read sequencing methods, also called synthetic long-read sequencing methods, work on the premise that they barcode long genomic DNA molecules prior to short-read sequencing, therefore retaining long-read information [181,182]. One such technology is 10× Genomics linked-read sequencing, which leverages microfluidics to partition and barcode high molecular weight DNA prior to short-read sequencing [181,182]. The second relatively newer option is the single tube long fragment reads (stLFR) method [183], that allows the barcoding of subfragments of long genomic DNA molecules via microbeads. It successfully barcodes subfragments in DNA molecules as long as 300 kb in length, with an average length of 50–70 kb [183]. Artificial long-read sequencing has the advantage over direct single-molecule long-read technologies in that it utilizes low error rate second-generation sequencing, which is much more cost-effective as well [181]. Although aLRS leverages many advantages of true long-read sequencing, some short-read issues still persist, e.g., PCR-bias and resolving intra-read complexity [171].

Compared to the traditional short-read NGS approaches, aLRS shows improved genome alignment and SV detection and allows haplotype reconstruction and phasing [184]. This technology also captures the “NGS dead zones” (i.e., highly homologous regions) with great improvement, including a net gain in read coverage in a total of 423 genes and 51 “dead zone” genes relevant to Mendelian disease [184]. This technology has been able to detect pathogenic variants in previously unsolved cases with NDs [185] but has also been useful in confirming and reconstructing complex pathogenic variants [181,184]. Last, it is also important to note that these aLRS techniques also are dependent upon the quality of DNA, and HMW DNA will allow the reconstruction of longer DNA fragments.

### 6.3. Optical Genome Mapping (OGM)

Optical genome mapping is a novel technique that fluorescently tags long linearized DNA molecules at specific sites to create a detailed map of genomic variation, including repetitive regions [186]. OGM allows the detection of insertions, deletions, translocations, balanced events (including inversions and balanced translocations), and more complex rearrangements and the detection of highly repetitive events, such as transposon-mediated pathogenic variants [186]. This method can identify SVs > 500 bp and up for both heterozygous and homozygous variants.

This technology requires ultra-high molecular weight (UHMW; molecules >150 kbp), and is, therefore, able to span, detect, and reconstruct very large and complex SVs. A limitation of this technology is that there is no sequence data is available; however, this method is complementary to sequence data and the combination of both can reconstruct highly complex SVs [172,187]. OGM has been shown to solve several genomic cold cases with ND/NND disorders [188,189], such as, for example, a mosaic complex of de novo deletion and inversion [188]. In addition, OGM has already entered the diagnostic space, including cytogenetics and molecular diagnostics. For example, with Bionano EnFocus™, they can accurately measure the number of *D4Z4* repeats in facioscapulohumeral muscular dystrophy (FSHD) [190].

### 6.4. Integrating Different Data Types

All these methods above are capable of de novo genome assembly and scaffolding, phasing, and the detection of large structural variants. Combining several methods, however, including short-read sequencing data, will provide an enhanced overview of the genomic variation in difficult-to-diagnose cases. This will allow researchers, for example, to identify bi-allelic variants for which both variants were identified using different techniques. In addition, if needed for variant reconstruction or to improve variant identification [187], hybrid scaffolding can be performed. The integration of two techniques, such as OGM and sequence data, has been shown to produce high-quality genomes [172,187].

## 7. Conclusions and Future Directions

In conclusion, a more comprehensive analysis of genomic cold cases can improve diagnostic rates, which can range from a more effective use of existing data to further experiments. In addition, AI applications can aid in both the initial data analysis and a well-timed re-analysis. However, human expertise will remain crucial in difficult-to-solve cases.

The emergence of novel sequencing and mapping technologies in genetic diagnostics has the ability to transform the diagnostic field, ranging from complex SVs to repeat expansion disorders, which are highly important, particularly in the field of neurology [191]. Using these novel techniques, we will be able to: (1) provide a more complete assessment of the human genome and genetic variation; (2) phase the genome into haplotypes, enabling the identification of maternal and paternal genetic variations; (3) assess variation in NGS “dead zones” (i.e., highly homologous regions); (4) more accurately assess and reconstruct structural variation; (5) more accurately evaluate repeat expansions; and (6) evaluate regions with high GC content. Overall, these techniques will improve our understanding of human variation and their involvement in disease [171].

The move towards LRS and OGM is limited by the quality of DNA, which may be challenging/impossible in already archived DNA biobanks. Although some size-selection methods are available to improve high-quality molecules in a DNA sample, it is imperative that the standard DNA isolation protocols shift towards the improved HMW and UWMW protocols and improved DNA handling methods to avoid shearing. Higher-quality DNA allows the identification and reconstruction of longer DNA fragments and the more optimal use of these technologies.

In conclusion, DNA sequencing and mapping technologies have accelerated at an unprecedented rate recently, and in the near future genome wide long-read approaches may become affordable to implement in a diagnostic setting. Improved diagnostic testing, which is crucial for therapeutic intervention and management, can bring the paradigm of personalized medicine in NDs one step closer.

## Data Availability

Not applicable.
